# Dense Ge nanocrystals embedded in TiO_2_ with exponentially increased photoconduction by field effect

**DOI:** 10.1038/s41598-018-23316-3

**Published:** 2018-03-20

**Authors:** A.-M. Lepadatu, A. Slav, C. Palade, I. Dascalescu, M. Enculescu, S. Iftimie, S. Lazanu, V. S. Teodorescu, M. L. Ciurea, T. Stoica

**Affiliations:** 1 National Institute of Materials Physics, 077125 Magurele, Romania; 2 University of Bucharest, Faculty of Physics, 077125 Magurele, Romania; 3 Academy of Romanian Scientists, 050094 Bucharest, Romania

## Abstract

Si and Ge nanocrystals in oxides are of a large interest for photo-effect applications due to the fine-tuning of the optical bandgap by quantum confinement in nanocrystals. In this work, dense Ge nanocrystals suitable for enhanced photoconduction were fabricated from 60% Ge in TiO_2_ amorphous layers by low temperature rapid thermal annealing at 550 °C. An exponential increase of the photocurrent with the applied voltage was observed in coplanar structure of Ge nanocrystals composite films deposited on oxidized Si wafers. The behaviour was explained by field effect control of the Fermi level at the Ge nanocrystals-TiO_2_ layer/substrate interfaces. The blue-shift of the absorption gap from bulk Ge value to 1.14 eV was evidenced in both photocurrent spectra and optical reflection-transmission experiments, in good agreement with quantum confinement induced bandgap broadening in Ge nanocrystal with sizes of about 5 nm as found from HRTEM and XRD investigations. A nonmonotonic spectral dependence of the refractive index is associated to the Ge nanocrystals formation. The nanocrystal morphology is also in good agreement with the Coulomb gap hopping mechanism of *T*^–1/2^ -type explaining the temperature dependence of the dark conduction.

## Introduction

TiO_2_ is a promising material for a broad area of applications such as photocatalysts^1–6^, dye sensitized and perovskite based solar cells^7–12^, rechargeable batteries^13^, gas sensors^14–16^ and biomedical devices^17^. However, its wide bandgap of about 3.2 eV represents a drawback for photovoltaic and photocatalytic applications, limiting the sensitivity to UV range. To extend the sensitivity in UV toward VIS, different methods have been proposed: doping or creation of trapping/recombination centres^2,18^; bandgap narrowing by reconstructing surfaces of TiO_2_^19,20^; deposition of a rough Au film with role of plasmonic sensitizer^21^; spun-cast deposition of Ge quantum dots (QDs) on TiO_2_ heterojunctions^22^.

A very promising solution for extending the photosensing of TiO_2_ toward VIS-IR is the fabrication of Ge-TiO_2_ nanocomposite with embedded Ge nanocrystals (NCs) or QDs. This solution has the advantage of a large exciton radius in Ge that enables a broad tuning of the electronic structure and consequently the spectral response of the Ge NCs/QDs based structures by a bandgap broadening with NC size decrease^23–27^. Examples are Ge NCs/QDs embedded in SiO_2_ for photosensing and light harvesting^28–33^. The electron confinement in QDs can improve the optical transition probability and can even lead to lasing effect as recently shown for Ge QDs in Si matrix^34^.

The reduction of the onset energy of the optical absorption in Ge-TiO_2_ nanocomposite by increasing the Ge content was already demonstrated^35,36^. Also, a photoconduction effect under white light illumination as well as a photovoltaic effect in the p-Si/ Ge-TiO_2_ heterojunction under AM1.5 solar illumination were shown to appear^35^. However, an in-depth analysis was not carried out in order to distinct between the contribution to the spectral photovoltage current of the light absorption in Ge NCs and in Si substrate. The influence of annealing temperature on the morphology and structure of Ge-TiO_2_ films deposited by magnetron sputtering with 50:50 Ge:TiO_2_ composition was previously studied by our group^37,38^. It was shown by annealing in a furnace that the amorphous state is preserved up to 500 °C, while for 600 °C and higher temperatures NCs of Ge and TiO_2_ are formed. However, the furnace annealing with inevitable low heating-cooling rates enhances the effect of the Ge diffusion toward surface, producing a significant loss of Ge. Therefore, in the present studies we used rapid thermal annealing (RTA) instead of furnace annealing for a better control of Ge crystallization, limiting the Ge diffusion effect^39^.

In this paper, we focus on the investigation of Ge NCs embedded in TiO_2_ obtained by magnetron sputtering deposition and subsequent RTA treatment. Our work adds results on new phenomena and understanding of the physics behind the properties of dense Ge NCs in TiO_2_ matrix by investigation of optical, electrical and photoelectrical properties in correlation with crystalline structure and morphology. Beside the expected quantum confinement effect of the blue shift of the optical bandgap in Ge NCs, other Ge NCs-related phenomena are evidenced on this composite material for the first time: a deep minimum in the spectral dependence of refractive index; Efros-Shklovskii *T* ^−1/2^ hopping conduction in the presence of a Coulomb gap in the electronic state distribution; spectral photocurrent enhanced by Ge NCs formation and carrier depletion induced by field effect.

## Results and Discussion

### Morphology and crystalline structure

The GeTiO_2_ as-deposited samples (Sasd) consist in a film of amorphous alloy of TiO_2_ and Ge as shown by XRD measurements (see below). The nanocrystallization occurs at the relatively low temperature of 550 °C. We comparatively present the morphology and structure of samples annealed at 550 and 700 °C, respectively (S550 and S700).

**Figure 1:**
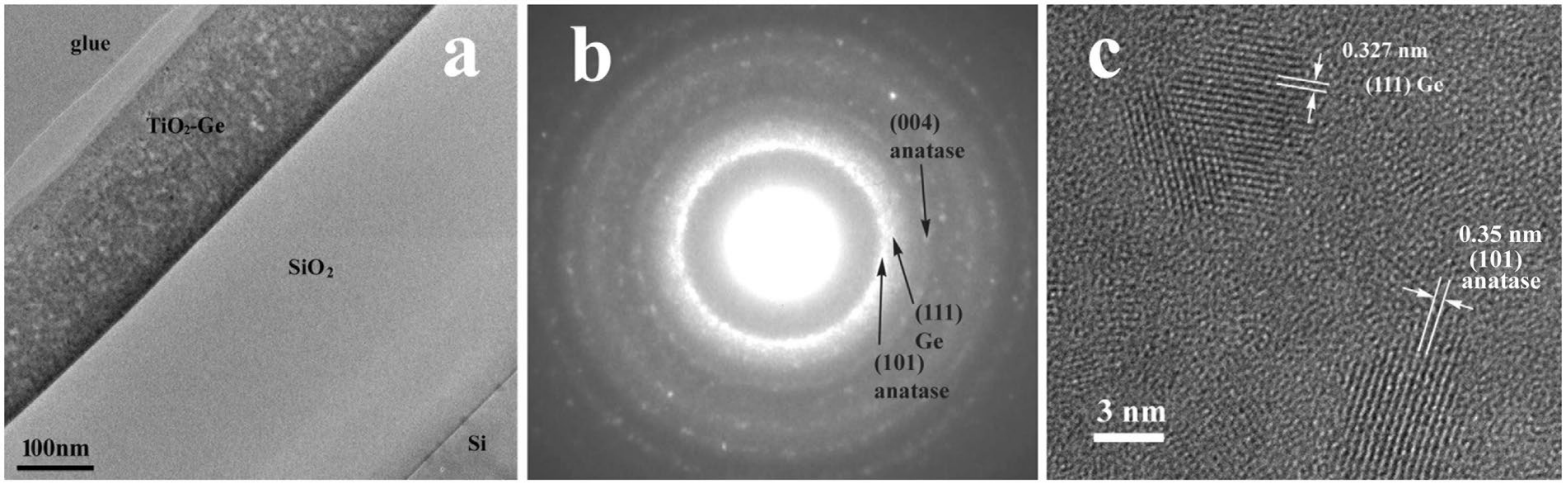
Sample S550 deposited on SiO_2_/Si substrate: (**a**) cross section TEM image, (**b**) SAED pattern and (**c**) HRTEM image showing anatase TiO_2_ and cubic Ge NCs.

Figure 1a presents the cross section TEM image of a sample S550 deposited on SiO_2_/Si substrate and annealed by RTA at 550 °C. The selected area electron diffraction (SAED) was performed from the TiO_2_-Ge film using a low diameter (150 nm) selected area. The SAED pattern evidences the presence of anatase TiO_2_ and cubic Ge NCs (Fig. 1b). The HRTEM image in Fig. 1c also clearly shows anatase TiO_2_ and dense (estimated to be 4 × 10^18^ cm^−3^) cubic Ge NCs with size of about 4–5 nm distanced from each other by 2–3 nm, having some amorphous phase in between them.

**Figure 2:**
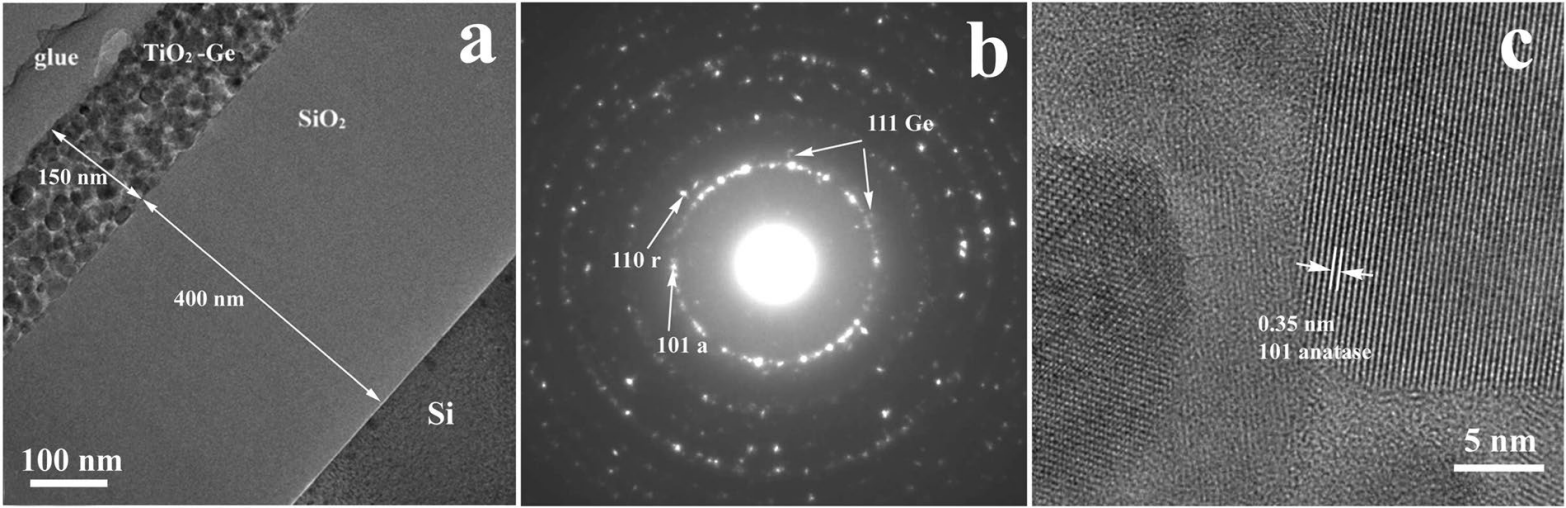
Sample S700 deposited on SiO_2_/Si substrate: (**a**) cross section TEM image showing the Ge-TiO_2_ film and SiO_2_ buffer; (**b**) SAED pattern showing TiO_2_ (anatase (101a) and rutile (110r) labelled spots) and very weak diffraction from cubic Ge (111); (**c**) HRTEM image showing the presence of anatase TiO_2_ NCs and amorphous areas between them.

The samples annealed by RTA at 700 °C, S700 are crystallized, but they show different structure and morphology than samples S550. Figure 2a presents the cross section TEM image at low magnification. The SAED pattern in Fig. 2b reveals the presence of TiO_2_ NCs, the most of them with anatase structure and a small part with rutile structure, as well as a very weak diffraction from cubic Ge NCs. The HRTEM image in Fig. 2c shows quite big well crystallized anatase TiO_2_ NCs with 20 nm average size (sizes between 15 and 40 nm).

**Figure 3:**
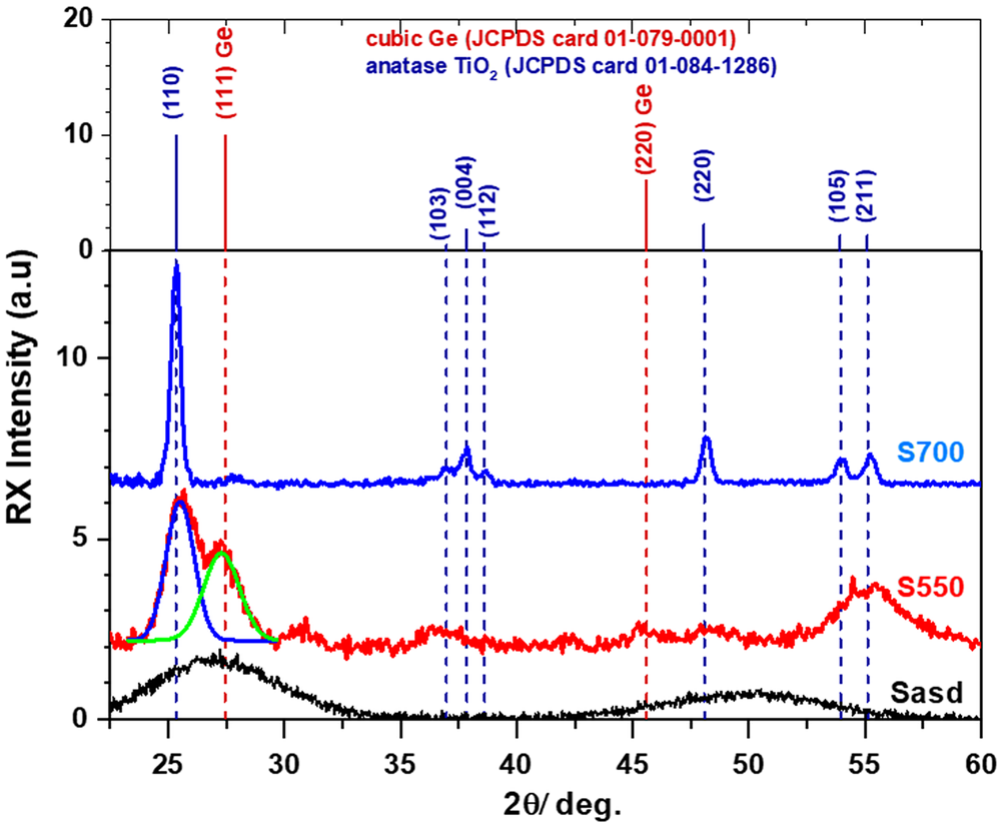
X-ray diffractograms of samples Sasd, S550 and S700 on 400 nm SiO_2_/c-Si substrate (smooth background was extracted). For S550: (111) Ge and (110) TiO_2_ peaks obtained from deconvolution are evidenced by green and blue curves. The topmost panel shows tabulated positions for cubic Ge and anatase TiO_2_.

The XRD and HRTEM results are in good agreement. Figure 3 shows the diffractograms of samples Sasd, S550 and S700 together with the standard tabulated positions of XRD peaks for cubic Ge and anatase TiO_2_. The sample Sasd shows amorphous structure with strongly broadened diffraction peaks. Sample S550 presents broad peaks corresponding to both NCs of cubic Ge and anatase TiO_2_ (deconvolution peaks). The Ge NCs mean size of 4.7 nm was obtained using the FWHM value of the (111) reflection peak. The sample S700 shows mainly anatase TiO_2_ peaks corresponding to TiO_2_ NCs with 20–30 nm size and very weak diffraction from cubic Ge. The low concentration of Ge NCs in sample S700 is explained by the strong diffusion and oxidation of Ge that occur at high temperatures^37^.

The structure and morphology results represent valuable information for optical, conduction and photoconduction properties as shown in the next sections. The obtained results on crystallization at low temperature (550 °C) for both Ge and TiO_2_ are important for reducing of thermal budget of layer fabrication for devices.

### Nanocrystallization effect on optical properties

**Figure 4:**
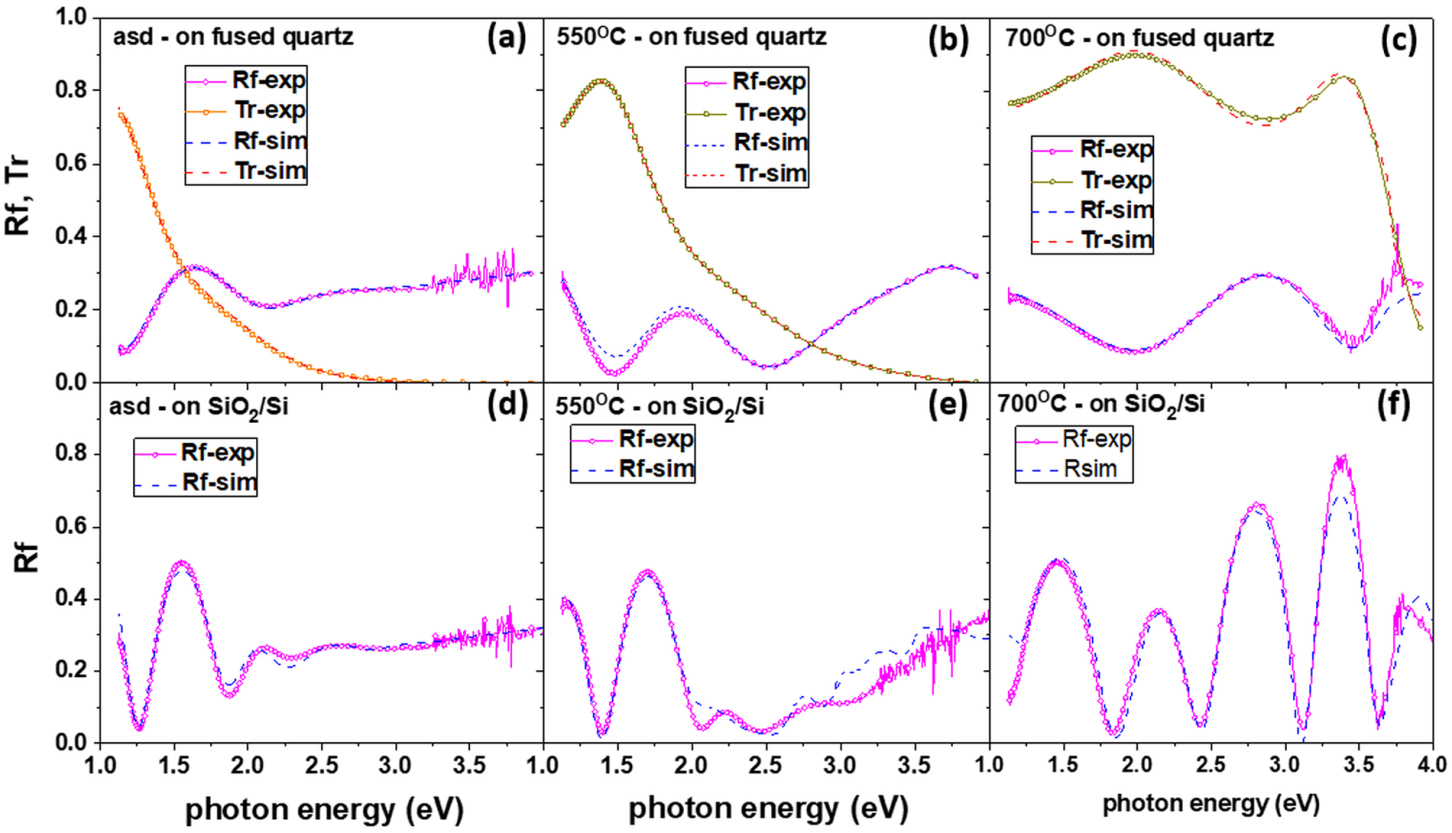
Optical properties of Ge-TiO_2_ layers - experimental (continuous lines with symbols) and simulated (dashed lines) spectra for samples Sasd, S550 and S700: (**a**–**c**) transmission and reflection of layers on fused quartz substrate and (**d**–**f**) reflectance of layers on SiO_2_/Si substrate.

Optical transmittance and reflectance were measured on films deposited on fused quartz substrates, namely on samples Sasd, S550 and S700 (Fig. 4a–c). For layers deposited on SiO_2_ (400 nm)/c-Si substrates, the samples being opaque due to Si substrate, only the reflectance spectra were measured (Fig. 4d–f).

A home-made software program has been used to simulate the spectral transmittance and reflectance of multilayer samples, using the transfer matrix formalism (e.g. ref.^40^). By fitting the simulated curves to the experimental transmission and reflection spectra, the dispersion curves of the refractive index (*n*) and extinction coefficient (*k*) have been computed.

The fit procedure uses analytic functions to describe the spectral dependence of the optical constants *n* and *k*:

1 $$k=\frac{\alpha \lambda }{4\pi }=\sum _{i=1}^{3}{b}_{i}{(E-{E}_{g,i})}^{m(i)}$$

2 $$n={n}_{0}+\sum _{j=1}^{3}{c}_{j}{E}^{j}$$

where: *α*, *λ* and *E* are absorption coefficient, wavelength and photon energy, respectively; *b*_*i*_ and *E*_*g*,*i*_ (bandgap) are parametric constants; *n*_0_ and *c*_*j*_ are parametric constants of Cauchy empirical formula; the exponent *m*(*i*) has two possible values of 2 and 1/2 for indirect (Tauc) and direct bandgap absorption, respectively^41^. This type of parametric fit was applied for samples Sasd and S700. For samples S550, a satisfactory fit using the simple analytic functions in eqs 1 and 2 was not possible. Therefore, in this case, a root-finder procedure was used for computing point by point the *n* and *k* pair that simultaneously ensures the equality of the computed and experimental transmittance and reflectance. The difficulty of such a procedure is related to the possible multiple solutions, especially for the region where the interference oscillations are stronger. This was overcome by a continuity condition in the energy dependence of the numerical solutions.

**Figure 5:**
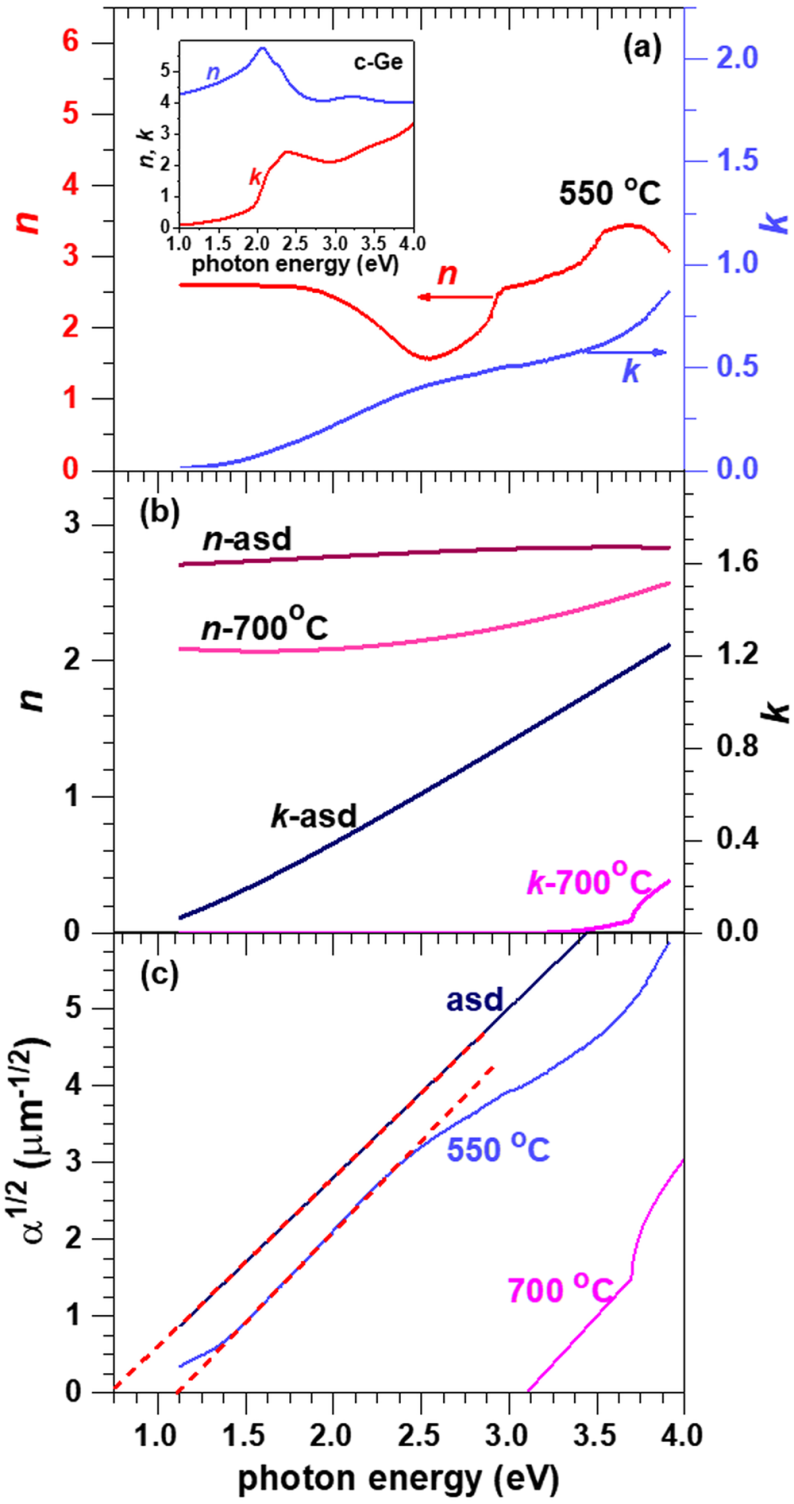
Optical constants *n* and *k* of: (**a**) sample S550 with Ge NCs (for comparison, the inset shows the curves of c-Ge based on tabular data^45^) and (**b**) samples Sasd and S700. (**c**) Tauc plot of *α* of samples Sasd, S550 and S700.

The spectral distribution curves of *n* and *k* coefficients for different annealing states are shown in Fig. 5a,b. A good agreement was obtained between computed and experimental transmission and reflection spectra for both fused quartz and SiO_2_/Si substrates, as can be seen in Fig. 4. The optical gap values *E*_*g*,*i*_ were estimated by linear extrapolation of the absorption coefficient *α* = 4*πk*/*λ* in Tauc representation (Fig. 5c).

As can be seen, the transparency of the layers constantly increases with the annealing temperature, resulting in the decrease and blue shift of the absorption coefficient.

For sample Sasd the absorption limit *E*_*g*_ is 0.73 eV, while for S550 the absorption threshold is about 1.14 eV in good agreement with the threshold of the spectral photocurrent as shown further. This blue-shift of the bandgap for Ge NCs in respect to bulk Ge can be explained by quantum confinement in NCs with diameter of about 5 nm in agreement with HRTEM and XRD results^26,27^. In comparison to our results on films with 60% Ge, smaller optical bandgap of about 0.7 eV was reported for smaller Ge content of only 33% in films deposited at 600 °C by sputtering from alloy GeTiO_2_ target^35^. An optical bandgap of about 2.0 eV was found in a Ge/TiO_2_ multilayer (Ge concentration of ~6%) deposited by ion beam sputtering and annealed in a furnace in air^36^. This spread of reported bandgap values demonstrates the strong dependence of the film properties on the preparation conditions that is reflected in different Ge NCs sizes and densities. Additionally, the NC shape and interface abruptness are also important for quantum confinement effects^42–44^. For S700, the absorption is almost cancelled in the VIS range and the optical gap of about 3.1 eV is close to that of a pure TiO_2_ film (Fig. 5c) in good agreement with the XRD and HRTEM results showing negligible Ge NCs density and dominant anatase TiO_2_. This can be explained by a strong diffusion of Ge to the layer surface that we showed to lead to the formation of tetragonal GeO_2_ at high temperature annealing^37^.

As shown in Fig. 5b, the refractive index of Sasd and S700 layers has smooth spectral dependence. In the case of S550, non-monotonic spectral dependence of the refractive index with a deep minimum at 2.5 eV associated with a slight saturation of the absorption band in Ge NCs in the 2.5–3 eV range was found (Fig. 5a). This result is related to the formation of Ge NCs. Such a minimum in *n*(*E*) is usually present at energies above a critical point (absorption limit), as in the case of bulk c-Ge where *n* decreases within the 2.0–2.5 eV range by about 1.5 units (inset of Fig. 5a)^45^. Less pronounced minimum was reported for the spectral dependence of the refractive index of Ge NCs formed on SiO_2_ surface^46,47^.

We can conclude that the nanocrystalline Ge-TiO_2_ composite obtained by annealing at 550 °C (S550) shows absorption in VIS-NIR, the absorption being lower than in the amorphous layer Sasd, but substantially enhanced in respect to TiO_2_. The estimated optical coefficients of the Ge-TiO_2_ composite films are important for the design and modelling of devices based on photosensing effects.

### Nanocrystallization effect on dark electric current

**Figure 6:**
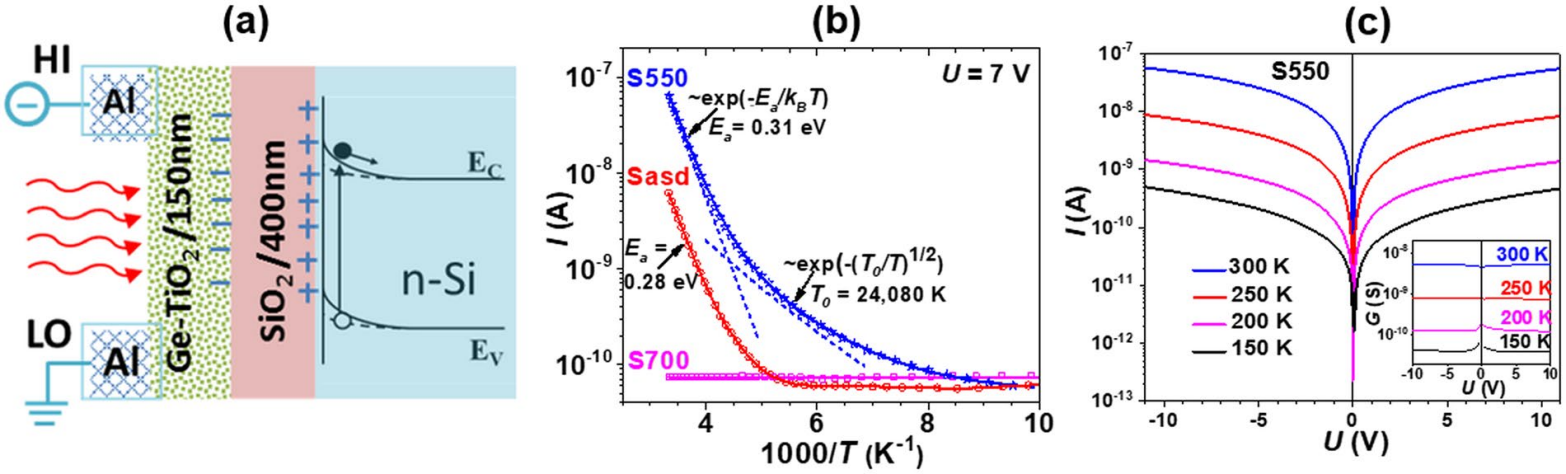
(**a**) Schematic of coplanar samples for dark and photocurrent measurements. (**b**) Experimental temperature dependence (curves with symbols) of the dark current for 7 V measured on samples Sasd, S550 and S700 and corresponding fit curves (continuous lines). For sample S550, the components of the fit curve are provided by the dashed lines representing the first and second terms in eq. 3, respectively. (**c**) The dark *I*–*V* dependence at different temperatures for sample S550. Inset shows the voltage dependence of conductance *G*.

Schematic of the Ge-TiO_2_/SiO_2_(400 nm)/Si samples for electrical and photoelectrical measurements is shown in Fig. 6a. The Si substrate was left as a floating gate electrode. The applied voltage *U* was varied from −20 V to +20 V corresponding to a small electric field in the Ge-TiO_2_ layer of up to 33 V/cm.

The distributed potential between electrodes generates an electric field in the SiO_2_ layer that can reach in our experiments a maximum value of 5.0 × 10^5^ V/cm, resulting in field effect induced charges at both SiO_2_/Si and Ge NCs-TiO_2_/SiO_2_ interfaces (Fig. 6a).

The dark current measurements as a function of temperature are shown in Fig. 6b for samples Sasd, S550 and S700. The samples show symmetric linear current-voltage (*I*–*V*) curves in the 150–300 K range, e.g. for sample S550 in Fig. 6c and inset. This means that phenomena related to possible rectifying junctions at contacts or those due to the charge accumulated by field effect at Ge NCs-TiO_2_/SiO_2_ interface have negligible effects on the dc current. However, the charged regions have significant contribution to the ac photocurrent (next section).

A fit of the temperature dependence of the dark current *I* for sample S550 was obtained by superposition of three components that perfectly describes the experimental data (Fig. 6b):

3 $$I=A{e}^{-\frac{{E}_{a}}{{k}_{B}T}}+B{e}^{-{(\frac{{T}_{0}}{T})}^{m}}+C$$

where *k*_*B*_ is the Boltzmann constant, *T* is the absolute temperature and the rest (*E*_*a*,_ *m*, *T*_0_ and *C*) are fit parameters. At high temperatures, the current is given by the Boltzmann component in equation (3) with activation energy *E*_*a*_ of about 0.30 eV. This component can be assigned to the transport of electrons thermally excited on delocalized states. The next term in equation (3) is given by a thermally activated hopping transport, with the exponent *m* = 1/2 and the value *T*_0_ of about 2.4 × 10^4^ K. Finally, at lower temperatures, the conductance has a contribution of tunnelling transport independent on temperature, namely the constant *C* in equation (3).

The value of the exponent *m* is very important for explaining the hopping mechanism. We found this parameter with a fit error smaller than 10%. The thermally activated hopping transport with exponent *m* = 1/2 is commonly reported in literature for NCs in layers of insulating ligands^48,49^. This behaviour was explained on the base of a “Coulomb gap”, i.e. a quadratic decrease in a localized state distribution at Fermi level. In the classical theory of variable range hopping (VRH) conductivity developed by Mott, the density of states distribution at Fermi level is taken as a constant. In Mott VRH, the coefficient *m* has the values dependent on the system dimensionality: *m* = 1/4, 1/3 and 1/2 for the dimensionality of 3, 2 and 1, respectively. However, Efros and Shklovskii (ES) demonstrated that the localized states distribution should vanish at Fermi level for an electronic system due to Coulomb interaction^50–52^. In this case, close to the Fermi level, inside the Coulomb gap, the state distribution is universally described by: *g*(*E*) ~ *E*^2^ for 3D system and *g*(*E*) ~ |*E*| for 2D system, where *E* is the energy relative to Fermi level. For both cases, under specific conditions depending on temperature and localized state system, there is a regime of hopping conductivity involving states from the Coulomb gap in which the temperature dependence of the conductivity *σ*(*T*) is described by ES-VRH:

4 $$\sigma (T)=B{e}^{-{(\frac{{T}_{0}}{T})}^{1/2}}$$

5 $${T}_{0}=\frac{f{e}^{2}}{{k}_{B}\varepsilon \xi }$$

where *B* is a constant parameter, *f* is a factor of the order of unity, *ε* is the effective dielectric constant of the assembly and *ξ* is the localization length^50^. With the localization length of the order of NC size, *ξ* ≅ 5 nm (Fig. 1c and Fig. 3), the effective dielectric constant of Ge-TiO_2_ nanocrystalline composite, *ε* ~ 20 and the experimental value of *T*_0_ = 2.4 × 10^4^ K (Fig. 6b) we evaluate the factor *f* to be about 11 that is close to the value given in^49,52^ for hopping in NCs systems.

At high temperatures, the thermal activation of carriers to extended states becomes the dominant conduction mechanism in the sample S550. The activation energy *E*_*a*_ has a value of 0.31 eV, close to the conduction band offset of Ge/TiO_2_ heterojunction^22,53^. The dominant thermal activation electronic transport over the hopping one demonstrates the potential of the transfer at room temperature (RT) of the photocarriers into TiO_2_ matrix. This is also important for applications based on photosensing effects in Ge-TiO_2_ nanocomposite.

The Ge NCs are absent in the amorphous Sasd sample and almost absent in the samples annealed at higher temperature S700. As a result, the conduction of these samples does not show ES hopping. As can be seen in Fig. 6b, the dark current in sample Sasd has only the components given by the thermal activation on extended states, exp(−*E*_*a*_/*k*_*B*_*T*) with activation energy *E*_*a*_ = 0.28 eV and by the temperature independent hopping on localized states of the amorphous structure (constant current *C*). Due to higher disorder, the extended-state mobility in sample Sasd is one order of magnitude smaller than that of the nanocrystalized sample S550. The sample S700 is a very resistive one in the measurement temperature range and only the tunnelling transport independent on temperature (component *C*) was observed (Fig. 6b).

### Nanocrystallization and field effect influence on spectral photocurrent

**Figure 7:**
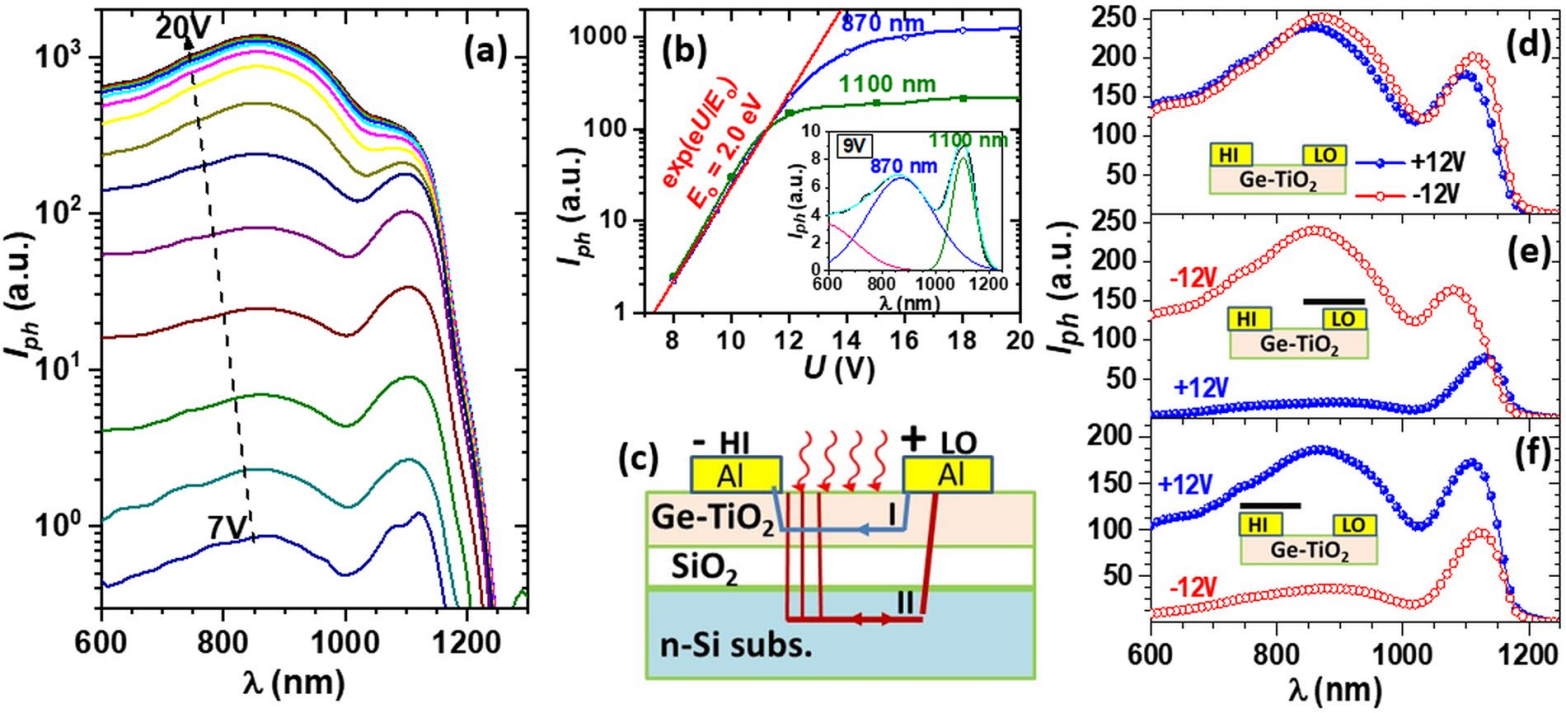
Photocurrent measurements on sample S550 of Ge NCs-TiO_2_/SiO_2_/Si (400 nm SiO_2_ thickness, 5 mm distance between electrodes). (**a**) Photocurrent spectra at RT for bias voltage varied in steps of 1 V. (**b**) Voltage dependence of the intensity of peaks at 870 nm and 1100 nm. Inset - deconvolution of spectrum for 9 V. (**c**) Schematic of samples and ac photocurrent paths for chopped light. (**d**–**f**) Comparison of the photocurrent spectra for +12 V and −12 V, for different shadowing of HI and LO electrode regions (schematic shown by insets): (**d**) without shadowing, (**e**) shadowed LO and (**f**) shadowed HI.

The spectral photocurrent was measured by illumination with modulated monochromatic light using lock-in amplifier technique, with the same coplanar geometry as for the dark current (Fig. 6a), with the Ge-TiO_2_ layer isolated from the substrate by a 400 nm thick SiO_2_ layer and the distance between electrodes of 5 mm. The photocurrent spectra of sample S550 with Ge NCs are shown for bias voltage varied between 7 and 20 V in Fig. 7a. The spectra show a broad peak at high photon energies (around 870 nm wavelength) due to photo-effects in Ge-TiO_2_ layer and a narrow peak at lower energies (about 1100 nm) due to surface photovoltage (SPV) and gating effects in crystalline Si substrate. The origin of the two contributions to the photocurrent spectra is discussed below. A broad photovoltaic spectrum with a wavelength threshold at about 1200 nm was reported for a Ge-TiO_2_(15%Ge)/p-Si heterostructure^35^, where a high contribution in the whole range of the spectrum can be due to the photo-effect in Si substrate. In our case, the Ge-TiO_2_ layer is isolated from the substrate by a 400 nm thick SiO_2_ layer.

The voltage dependence of the intensities of the main peaks at 870 nm and 1100 nm (Fig. 7b) are obtained by deconvolution. An example of deconvolution is illustrated in the inset of Fig. 7b. The photocurrent due to photo-effects in Ge-TiO_2_ layer with the peak at 870 nm has the threshold at about 1100 nm (~1.14 eV) in good agreement with the optical bandgap value found from optical absorption studies (Fig. 5c). To describe the tail of the signal from low wavelengths, which is not well resolved within the experimental range, an additional broad peak at wavelength smaller than 600 nm had to be considered. As we can see in Fig. 7b, the intensity of both maxima at 870 nm and 1100 nm varies exponentially for low voltages as *I*_*ph*_ ~ exp(*eU*/*E*_0_), and differently saturates: the peak at 1100 nm saturates earlier for voltage *U* > 12 V, while the broad one at 870 nm continues to increase up to above 15 V. The ac photocurrent produced by a chopped light illumination (frequency of 120 Hz) may have beside the path through the Ge-TiO_2_ layer (path I), an important component through the Si substrate by capacitive coupling (path II), as schematically shown in Fig. 7c. The electric field in SiO_2_ between Ge-TiO_2_ layer and Si substrate induces the charging of the Ge-TiO_2_ layer. This field effect enhances the photoconduction by creation of a depleted zone in the layer with lower dark carrier concentration and higher photosensitivity (see Fig. 6a and discussion below). This result shows the importance of the Fermi level control by field effect or doping for the increase of the photo-effect. Additional contribution to the photocurrent at wavelengths near Si bandgap due to the substrate influence (peak at 1100 nm) is given by SPV and gating effect^54–57^.

The non-linear behaviour of the photocurrent intensity (Fig. 7b) is in contrast to the linear dependence of the dark current (Fig. 6c). Possible phenomena explaining non-linear voltage dependence of the photocurrent are the field assisted thermal ionization of electron-hole pairs photo-generated inside NCs and a heterojunction effect at Ge NCs/TiO_2_ interfaces. For such effects, a high local electric field is necessary. This is possible only in the charged regions of the junction Al/Ge-TiO_2_, or at Ge-TiO_2_/SiO_2_ interface (Fig. 6a), the average value of the electric field parallel to the Ge-TiO_2_ layer being quite low, of up to 33 V/cm.

In order to investigate the influence of the contact regions we have measured photocurrent spectra by shadowing either HI or LO contact regions. The results are illustrated in Fig. 7d–f. Without shadowing any contact, the measured spectra are almost the same for positive and negative 12 V polarization of the HI contact (Fig. 7d). For shadowing of the LO contact region (Fig. 7e), the photocurrent intensity for the HI negative bias of −12 V (hole depletion in the illuminated zone of the Ge-TiO_2_ layer) is much higher than for positive +12 V one (hole accumulation in the illuminated zone of the layer). On contrary, the photocurrent for HI positive voltage is higher for shadowing the HI contact region (Fig. 7f). From these results, it is quite clear that the region of the negatively biased contact has the major contribution to the photocurrent. The charged regions at both Al/Ge-TiO_2_ and Ge-TiO_2_/SiO_2_ interface may contribute to the non-Ohmic photocurrent by the effect of the internal electric field of the charge region (field assisted thermal ionization), as well as by changing the electron population of Ge NCs (changes of conductance and carrier recombination properties). Thus, the exponential increase of the photocurrent as a function of the bias voltage can be explained by hole depletion zone induced by field effect.

For deeper investigation of the substrate influence on spectral photocurrent through field effect and its associated SPV and gating effects we additionally performed measurements on nanocrystallized Ge-TiO_2_ layers using samples with different thicknesses of the SiO_2_ buffer layer on Si substrate and different distances between the coplanar electrodes. The Ge-TiO_2_ deposition and RTA parameters are those of the sample S550.

**Figure 8:**
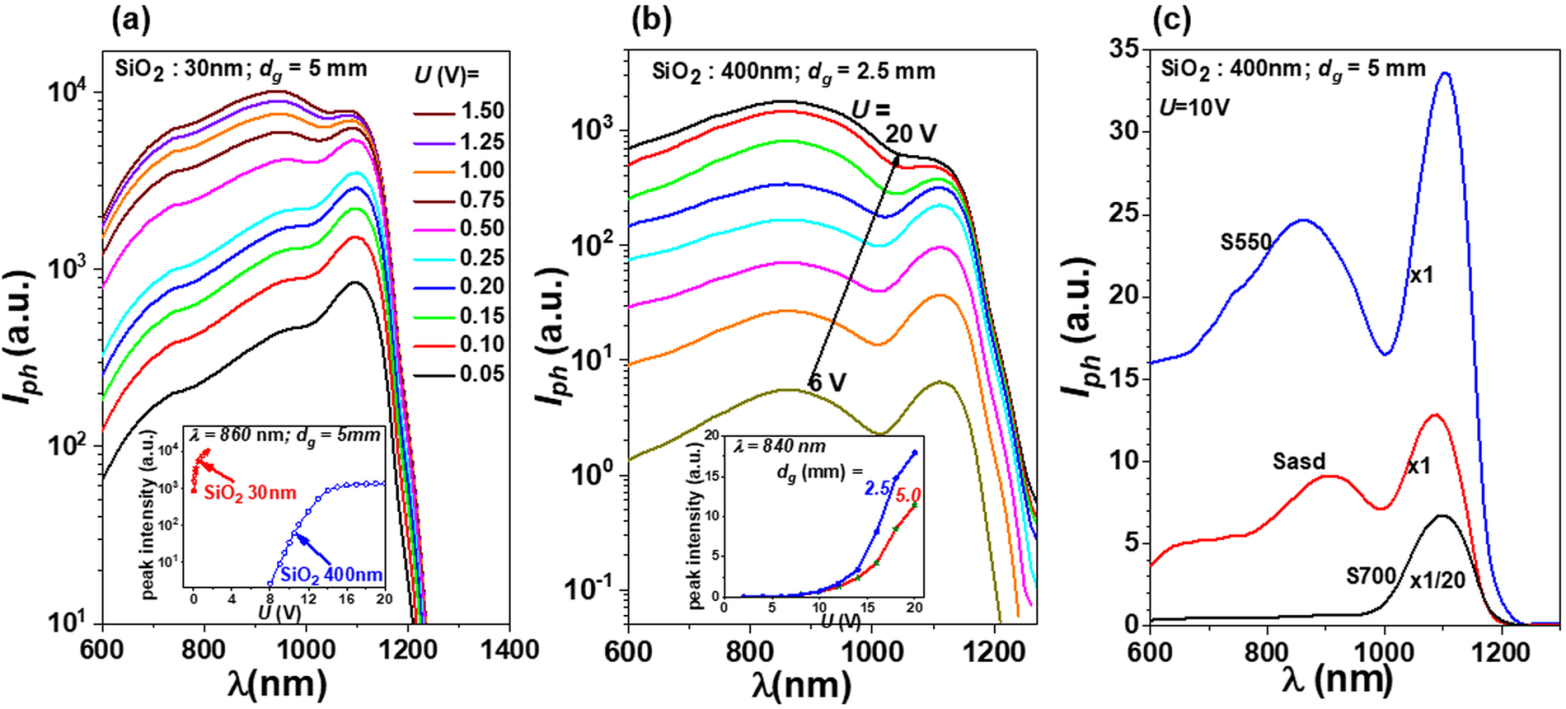
Spectral photocurrent at RT for samples with Ge NCs in TiO_2_ of S550 layers: (**a**) sample with SiO_2_ buffer thickness of 30 nm (the inset shows the comparison of the voltage dependence of the peak intensity for 30 nm and 400 nm buffer thickness); (**b**) sample with smaller gap between electrodes *d*_*g*_ of 2.5 mm – bias voltage varied in steps of 2 V (the inset shows the comparison of the voltage dependence of the peak intensity for *d*_*g*_ of 2.5 mm and 5.0 mm); (**c**) comparison of the spectral photocurrent at RT for samples Sasd, S550 and S700.

The spectra measured on sample with thinner SiO_2_ buffer of about 30 nm instead of 400 nm (Fig. 8a) show similar behaviour as those in Fig. 7a, but have higher intensity at smaller values of the applied voltage. The voltage dependence of the peak intensity at 870 nm wavelength for 30 nm SiO_2_ buffer is compared in the inset of Fig. 8a with the dependence found for the 400 nm buffer. For thinner SiO_2_ buffer of 30 nm, the intensity starts to saturate at applied voltage of about 0.5 V, much smaller value than that of 15 V for the case of 400 nm SiO_2_ buffer. The saturation value of photocurrent for the 30 nm SiO_2_ buffer is found to be higher with more than one order of magnitude than that for 400 nm SiO_2_ buffer. This result also demonstrates the photocurrent enhancement, more pronounced for thinner SiO_2_ buffer.

The reduction of the gap *d*_*g*_ between electrodes from 5 mm to 2.5 mm has little effects on the shape of the photocurrent spectra and the voltage dependence, as can be seen in Fig. 8b in comparison to the curves in Fig. 7a. As expected, the photocurrent increases by reduction of *d*_*g*_, the peak intensity reaching a 50% higher value for 20 V applied voltage (inset of Fig. 8b). The non-linear dependence of the photocurrent as a function of applied voltage and the gap between electrodes has the origin in non-linear contributions due to field effects.

Finally, we present the influence of the annealing temperature on the photocurrent spectra. In Fig. 8c, the photocurrent spectra for 10 V measured at RT on samples Sasd, S550 and S700 are presented. Under same measurement conditions, the spectral photocurrent of the nanocrystalline sample S550 is higher than that of the amorphous sample Sasd. As discussed above, the spectra show a broad peak at high photon energies (corresponding to 860–910 nm wavelengths) due to photo-effects in Ge-TiO_2_ layer and a narrow peak at lower energies (about 1100 nm) due to SPV and gating effects in crystalline Si substrate. For sample S700, the spectral photocurrent is dominated by the peak at 1100 nm attributed to the SPV effect of the substrate. This peak is very much increased in this case due to the high transparency of the Ge-TiO_2_ layer (shown in section *Nanocrystallization effect on optical properties*), while the broad peak is almost cancelled because of the low concentration of Ge NCs (see the results in section *Morphology and crystalline structure*).

To conclude, the results show the importance of the Fermi level control by field effect or doping for the increase of the photo-effect in Ge NCs-TiO_2_ films.

## Conclusions

The optical, electrical and photoelectrical properties of Ge-TiO_2_ nanocomposite are correlated with the nanocrystallization of Ge. The layers of 60% Ge in TiO_2_ were prepared on SiO_2_/Si substrates by magnetron sputtering deposition and subsequent RTA. The as-deposited amorphous layers show the optical bandgap of 0.73 eV. RTA at 550 °C results in the formation of Ge NCs embedded in TiO_2_, with diameter of about 5 nm and a blue shift of the optical bandgap to 1.14 eV due to quantum confinement effect in Ge NCs. The refractive index spectrum of this layer shows a minimum at about 2.5 eV associated with Ge NCs formation. For annealing at higher temperature of 700 °C, the layers show big TiO_2_ NCs and very low Ge NC density due to fast diffusion of Ge and its oxidation. This result explains the negligible absorption in VIS-NIR range and optical bandgap shifted to about 3.1 eV.

The structure and optical properties are well correlated with the dark and photocurrent properties of the layers. In the sample with Ge NCs formed by RTA at 550 °C, the dark conduction at RT is dominated by the activation of carriers to extended states over the Ge NCs/TiO_2_ barrier of 0.3 eV. Efros-Shklovskii *T*^–1/2^ variable range hopping on states in parabolic Coulomb gap was revealed at intermediate temperatures, that is in agreement with 4–5 nm size of Ge NCs. The spectral photocurrent depends on the annealing temperature. The photocurrent spectra show a broad peak at 860–910 nm attributed to the photo-effect in Ge-TiO_2_ layer (as-deposited and 550 °C RTA, only) and a narrow peak at about 1100 nm due to surface photovoltage in crystalline Si substrate. The 700 °C RTA sample with very low Ge NC density shows a very poor photoconduction of the layer. The photoconduction of 550 °C RTA samples is one order of magnitude higher than that of as-deposited sample due to the film nanocrystallization. Additionally, an exponential increase of the photocurrent in Ge NCs due to the carrier depletion zone induced by field effect is demonstrated. Moreover, results obtained on samples with different SiO_2_ buffer thicknesses and different gaps between electrodes are in agreement with photocurrent enhancement induced by field effects. The results reported in this paper pave the way for photo-effects applications by offering solutions for enhancing the photoelectric activity of Ge-TiO_2_ films in VIS-NIR.

## Methods

### Preparation of Ge-TiO_2_ nanocrystalline composites

Films of GeTiO_2_ alloy were obtained by co-sputtering deposition of Ge and TiO_2_ on fused quartz and n-type c-Si substrates in a magnetron sputtering equipment (Surrey Nanosystems, Gamma 1000) using pure Ar (6 N) atmosphere at 4 mTorr pressure. DC and RF plasma were employed for Ge and TiO_2_ sputtering, respectively. The c-Si substrates have been covered before deposition with a SiO_2_ layer of ~400 nm thickness. The GeTiO_2_ as-deposited films (Sasd) have about 180 nm thickness and a Ge volume concentration of 60%. Samples with SiO_2_ buffer layer of 30 nm were also investigated. The samples Sasd were annealed for 10 min in Ar atmosphere at 550 °C and 700 °C by RTA (Annealsys AS-Micro) in order to obtain Ge-TiO_2_ nanocrystalline composites, the annealed samples being called in the paper samples S550 and S700.

### Characterization

The morphology and structure of layers were investigated by high resolution transmission electron microscopy (HRTEM, Jeol ARM 200 F electron microscope). X-ray diffraction (XRD) measurements were performed on a D8 Advance X-ray diffractometer (Bruker). Optical transmission and reflectance spectra were measured within the 300–1100 nm range (Cary 5000 version 1.12 and Lambda 45 UV/Vis spectrophotometers). For electrical and photoelectrical measurements, coplanar samples were obtained by thermal evaporation of Al electrodes with two gaps of 5.0 and 2.5 mm between them. Dark and photocurrent measurements were performed in vacuum in a cryostat with He closed-circuit cooling system. The dark current was recorded using a Keithley 236 Source Measure Unit. For spectral photocurrent measurements in the wavelength range from 600 to 2000 nm, a setup consisting in a halogen 50 W lamp, a SR540 optical chopper, a grating monochromator Newport Cornerstone™ 260 with 550 nm and 1000 nm longwave pass filters, Stanford SR810 lock-in amplifier and Keithley 236 Source Measure Unit was employed. The photocurrent spectra are obtained in a.u. by normalizing the measured photocurrent to the spectrum of the lamp.

### Simulation

A home-made software is implemented to conduct all the optical simulations. The transfer matrix formalism is used considering the layered structure of investigated samples, Ge-TiO_2_/SiO_2_/Si.
